# Effect of Pregnancy on Quantitative Medication Use and Relation to Exacerbations in Asthma

**DOI:** 10.1155/2017/8276190

**Published:** 2017-07-20

**Authors:** So-My Koo, Yunsun Kim, Chorong Park, Gun Woo Park, MoonGyu Lee, Sungho Won, Hyeon-Jong Yang

**Affiliations:** ^1^Division of Allergy and Respiratory Medicine, Department of Internal Medicine, Soonchunhyang University Seoul Hospital, Soonchunhyang University College of Medicine, Seoul, Republic of Korea; ^2^SCH Biomedical Informatics Research Unit, Soonchunhyang University Seoul Hospital, Seoul, Republic of Korea; ^3^Department of Public Health Science, Graduate School of Public Health, Seoul National University, Seoul, Republic of Korea; ^4^Interdisciplinary Program of Bioinformatics, Seoul National University, Seoul, Republic of Korea; ^5^Institute of Health and Environment, Seoul National University, Seoul, Republic of Korea; ^6^Pediatric Allergy and Respiratory Center, Department of Pediatrics, Soonchunhyang University Seoul Hospital, Soonchunhyang University College of Medicine, Seoul, Republic of Korea

## Abstract

**Background:**

The quantification of asthma medication reduction and its relation to an aggravation of asthma during pregnancy at an individual level are unclear.

**Methods:**

We conducted a nationwide retrospective cohort study of asthmatic pregnant women in South Korea. All of the asthma medications were ranked from 1 to 4 according to the guideline-based stepwise approach. We assessed the daily sums of the ranks of the asthma medications and their association with exacerbations during three phases based on the individual's delivery date: before, during, and after pregnancy.

**Results:**

The study cohort included 115,169 asthmatic pregnant women who gave birth between 2011 and 2013. The subjects were clustered into four groups according to the daily rank sums of their asthma medication. Asthma medications were rapidly reduced at the beginning of the pregnancy and then slowly increased after delivery. Exacerbations were more frequent in the group with higher rank-sum values than in the group with lower values. Overall exacerbations were reduced during pregnancy compared to before or after delivery.

**Conclusions:**

Asthmatic pregnant women tended to reduce their asthma medication use during pregnancy. This led to a greater number of exacerbations in a small part of the study population.

## 1. Introduction

Pregnancy affects the severity and control of asthma and is one of the most important risk factors for the exacerbation of asthma [1]. The prevalence of asthma during pregnancy is known to be 3%–12% [2, 3], and the rate of women requiring medical intervention due to exacerbation during pregnancy is about 20% [4]. It is well known that the maintenance of asthma therapy during pregnancy outweighs the risk to the fetus [5, 6]. Poorly controlled asthma can have a negative effect on pregnant women and fetuses. Asthma exacerbations increase the risk of a preterm delivery, low birth weight, perinatal mortality, and preeclampsia [1, 7]. Indeed, several reports have shown that fetal anomalies are likely to be associated with asthma exacerbations rather than the use of asthma medication during pregnancy [8, 9], and international guidelines consistently recommend the continuation of asthma medication during pregnancy. However, despite this, most studies have consistently reported that asthmatic women during pregnancy tend to stop or switch their asthma medication due to concerns regarding adverse effects on the fetus [10–14]. Some studies have suggested that the severity of asthma increases the exacerbation rate [4, 15], and the severity of asthma before pregnancy is related to subsequent exacerbations during pregnancy and asthma symptoms during labor and delivery [4]. Therefore, severe asthma should be closely monitored and adequately controlled during pregnancy.

It is well known that one-third of pregnant women with asthma experience worsened symptoms, another third improve, and the others show no change [16, 17]. Stopping or poor compliance with asthma medication may aggravate asthma in pregnancy. On the other hand, previous severe asthma can nevertheless aggravate asthma symptoms without the patient stopping their asthma medication, and naturally improved asthma during pregnancy may be well controlled despite discontinuing with asthma controllers. Most of the previous studies have, however, investigated patterns of prescriptions and asthma symptoms at the population level but not at the individual level. To the best of our knowledge, no studies have investigated changes at the level of asthma medications and their correlations with asthma exacerbations before, during, and after pregnancy at the individual level. For this reason, further study is needed to evaluate the effect of pregnancy on medication use and its correlation with asthma exacerbation at the individual level. We hypothesized that asthmatic women tend to reduce or stop the use of asthma medication during pregnancy and that the correlation with asthma exacerbation will differ according to their severity of asthma and its natural course before and during pregnancy. To prove this hypothesis, we conducted a nationwide population-based cohort study to evaluate the quantitative changes in asthma medication during three phases of pregnancy (before, during, and after pregnancy) and their correlation with asthma exacerbation.

## 2. Methods

### 2.1. Data Sources

The Health Insurance Review and Assessment Service (HIRA; Wonju, Republic of Korea), a government-affiliated agency responsible for examining the accuracy of claims for National Health Insurance and National Medical Aid in South Korea, covers approximately 96.6% of the South Korean population [14]. The HIRA database includes reviewed information regarding demographics, medical healthcare service data including a set of diagnostic codes (International Statistical Classification of Disease and Related Health Problems, 10th Edition; ICD-10), dispensed prescribed medications, and diagnostic tests and procedures. In particular, it includes all information on hospital stays, emergency department (ED) visits, and outpatient clinic visits. Missing or aberrant values in key fields such as drug name, quantity, date dispensed, and duration comprise under 0.5% of all records [18]. We used the HIRA database to conduct a nationwide population-based observational retrospective cohort study of asthmatic pregnant women.

### 2.2. Study Subjects and Design

**Figure 1:**
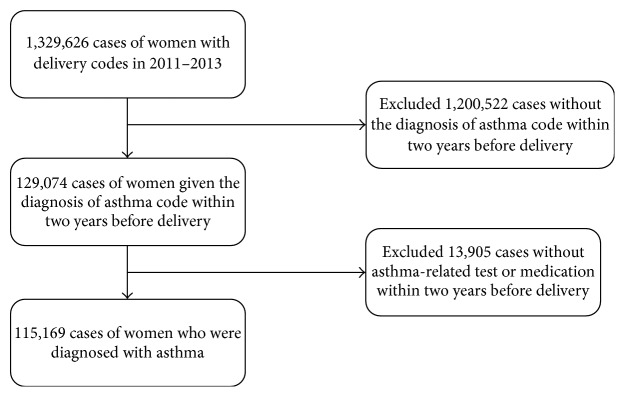
Flow diagram of the study cohort.

**Figure 2:**
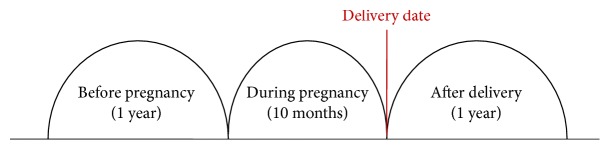
Schematic diagram of the three phases of observation.

A total of 1,329,626 women assigned a delivery code were identified by a review of the HIRA data for the period of January 1, 2011, to December 31, 2013. Among them, 115,169 asthmatic pregnant women were identified who met all of the following criteria: (1) a delivery between January 1, 2011, and December 31, 2013; (2) a diagnosis code for asthma according to the ICD-10 J45.x-J46.x code set within two years before the delivery date; and (3) being prescribed asthma medication or undergoing diagnostic tests for asthma at least once within two years before the delivery date (Figure 1). Detailed information regarding asthma medications is described below, and diagnostic tests for asthma included spirometry with or without a bronchodilator response and bronchial provocation tests. The observation period was divided into three phases: 1 year (365 days) before pregnancy, during pregnancy (280 days) before the delivery date, and 1 year (365 days) after pregnancy. The delivery dates were determined from the procedure codes related to delivery. Daily asthma medications, asthma exacerbations, and healthcare utilization were observed from one year before pregnancy to one year after delivery (Figure 2).

### 2.3. Asthma Medications and Their Quantitative Rank

The asthma medications were defined as inhaled corticosteroids (ICSs), ICS combined with inhaled long-acting *β*_2_-agonists (ICS/LABAs), inhaled short-acting *β*_2_-agonists (SABAs), LABAs, long-acting muscarinic antagonists (LAMAs), oral leukotriene receptor antagonists (LTRAs), xanthine derivatives, and systemic corticosteroids. Daily asthma medications, based on the codes for prescribed and dispensed medications, were captured through the three phases of pregnancy and were ranked from the level of controller as classified by the Global Initiative for Asthma guidelines with respect to the stepwise approach [19]. Low-dose ICSs [20–23], LTRAs [24], xanthine [25], or LABAs [26] were defined as rank 1, medium- to high-dose ICS [27, 28] or low-dose ICS/LABA combination [29] inhalers as rank 2, and medium- to high-dose ICS/LABA combination [30] inhalers as rank 3. Rank 4 was defined as any of the following: (1) LAMA [31] inhaler and (2) long-term use of oral prednisolone of less than 20 mg or other types of corticosteroids [32] with the same potency (betamethasone at 2.4 mg, dexamethasone at 3 mg, or methylprednisolone at 16 mg) (Table 1). We calculated the daily rank of asthma medications at the individual level. If subjects took more than or equal to two different asthma medications at the same time, the sums of their ranks were added up to a maximum of rank 4. ICSs were marked as their ranks for each consecutive day they were taken (e.g., LTRA and low-dose ICS were ranked as 1 and 60 doses of ICS used twice daily ranked as 1 for 30 days). The standard ICS/LABA inhaler contains 60 doses for use over 30 days (Table 1). The rank-sum values of the asthma medications were plotted for each patient to indicate time-varying patterns. Corticosteroid burst therapy and SABAs were not ranked but were defined as a mark of asthma exacerbation.

### 2.4. Definition of Asthma Exacerbations

Asthma exacerbations were defined as one of the ICD-10 asthma codes along with urgent events satisfying the following conditions occurring on the same date: asthma-related outpatient clinic visit with systemic corticosteroid burst, as mentioned above (i.e., more than 20 mg of prednisolone or other types of corticosteroids with the same potency); asthma-related hospitalization; asthma-related ED visit; or outpatient clinic visit with SABA nebulizer treatment under the ICD-10 asthma codes.

### 2.5. Healthcare Utilization

The daily records relating to healthcare use were reviewed and classified as hospital stay, ED visit, and outpatient clinic visit. Furthermore, outpatient clinic visits were subdivided according to their specialty, for example, internal medicine, obstetrics/gynecology, general practitioner, general surgery, otolaryngology, and family medicine.

### 2.6. Ethical Considerations

The research protocol for this study was approved by the Ethical Review Board of Soonchunhyang University Seoul Hospital (approval number: SCHUH 2016-04-024).

### 2.7. Definition of Clustered Groups

Study subjects were clustered into groups according to their pattern of asthma medication using the statistical methods detailed below.

### 2.8. Statistical Analyses

Rank-sum values of the asthma medications for each subject were calculated every month and the overall trends during that time were visualized with a spaghetti plot. Additionally, the proportions of subjects who experienced asthma exacerbations for one year before, during, and one year after pregnancy were also calculated. In total, subjects were followed up for 1,010 days. Continuous variables, such as the prescribed and dispensed amounts of asthma medications and the number of asthma exacerbations and healthcare utilization, are presented as means ± standard deviations. Monthly rank-sum values of the asthma medications for one year before, during, and one year after pregnancy were calculated for each subject and compared with a multivariate analysis of variance to detect whether asthma medication use was affected by pregnancy. The rank-sum values were also used for clustering with the *k*-means algorithm. The *k*-means algorithm was performed using Euclidean distances calculated from three rank-sum values for one year before, during, and one year after pregnancy and the number of clusters was determined by *r*^2^, which is defined as the “sum of squares between” over the “sum of squares total.” These analyses were conducted with PROC FASTCLUS and subjects were clustered into four groups. For each group, all statistical analyses were conducted to detect the pattern of drugs related to asthma exacerbations.

Associations between the daily rank-sum values of the asthma medications and asthma exacerbations were analyzed with a quasi-likelihood-based approach. Analyses were conducted with PROC GLIMMIX (SAS version 6.1). For each subject, the number of asthma exacerbations was counted during pregnancy and was used as a response variable. It should be noted that the response variables have very large values and were thus assumed to follow a quasi-Poisson distribution. The logarithm was used as a link function, and this will be referred to as a quasi-Poisson regression for the remainder of this report. The rank-sum values of the asthma medications for one year before, during, and one year after pregnancy were centered with their global sample mean and were used as explanatory variables. We let *Y*_*i*_ be the number of asthma exacerbations of subject *i*. If we denote the centered rank-sum values of the asthma medication before, during, and after pregnancy by *X*_*i*1_, *X*_*i*2_, and *X*_*i*3_, respectively, the quasi-Poisson regression is

(1) log⁡EYi=β0+β1Xi1+β2Xi2+β3Xi3+β4Xi1Xi2+β5Xi2Xi3+β6Xi1Xi3i:  independent  andvar⁡Yi=c×EYi.

We considered two-way and three-way interactions of rank-sum values, and a stepwise selection method was used to find the best model. Estimated regression coefficients and 95% confidence intervals are presented. All statistical procedures were conducted using the SAS Enterprise statistical software (version 6.1). The alpha level for the determination of significance was 0.10.

## 3. Results

### 3.1. Daily Rank-Sum Values of Asthma Medications before, during, and after Pregnancy

**Figure 3:**
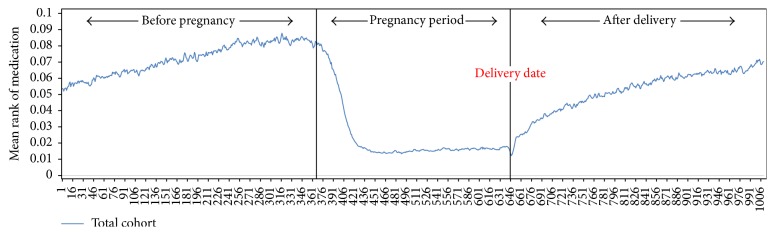
Daily rank-sum values of asthma medications during the time periods. The overall rank-sum value of the asthma medications tended to be abruptly reduced at the beginning of pregnancy compared with before and slowly increased after pregnancy.

**Figure 4:**
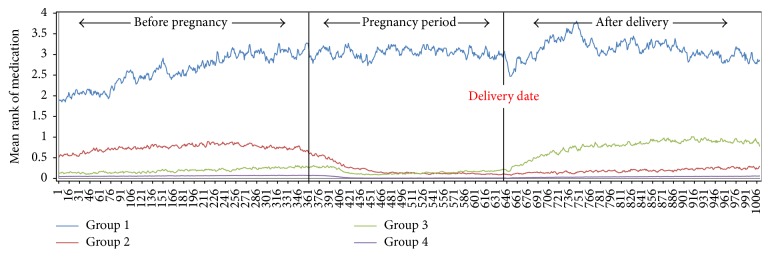
The 115,169 asthmatic pregnant women were clustered into four distinct groups. Group 1 (*n* = 225, severe persistent group) showed the highest level of daily rank-sum values of asthma medications. Group 2 (*n* = 3,251, improved asthma group) showed an abrupt decreasing trend of rank-sum values during pregnancy and maintained a stable rank-sum value after pregnancy. Group 3 (*n* = 2,968, worsened asthma group) showed an abrupt increasing trend after delivery. Group 4 (*n* = 108,725) did not show any particular change in trend.

The overall rank-sum value of the asthma medications tended to be abruptly reduced just after women became pregnant and slowly increased after pregnancy (Figure 3). Study subjects were clustered into four groups according to the pattern of their asthma medication. Group 1 (*n* = 225) showed the highest level of daily rank-sum values of asthma medications, with many spikes over the study period. Group 2 (*n* = 3,251) showed the second highest level of rank-sum values before pregnancy and showed an abruptly decreasing trend during pregnancy. Group 3 (*n* = 2,968) showed a small decreasing trend during pregnancy compared to before pregnancy and an abruptly increasing trend after delivery. While Group 2 maintained a similar level of rank-sum values during and after pregnancy, Group 3 showed higher rank-sum values after pregnancy than during it. Group 4 (*n* = 108,725) did not show any particular change in trend (Figure 4).

### 3.2. Patterns of Asthma Medications before, during, and after Pregnancy

The total amounts of asthma medications dispensed are shown in Tables 2 and 3. All of the oral medications including LTRAs, xanthine, LABA, and systemic corticosteroids as a controller dose showed the consistent result that oral asthma medications were less used during pregnancy than before and after pregnancy (Table 2). This finding was consistently observed regardless of group. Otherwise, inhaled asthma medications showed contradictory patterns in that low-to-medium doses of ICSs were used more during pregnancy than before and after it, while other inhaled asthma medications showed similar patterns to the oral ones (Table 3).

### 3.3. Asthma Exacerbations before, during, and after Pregnancy

**Figure 5:**
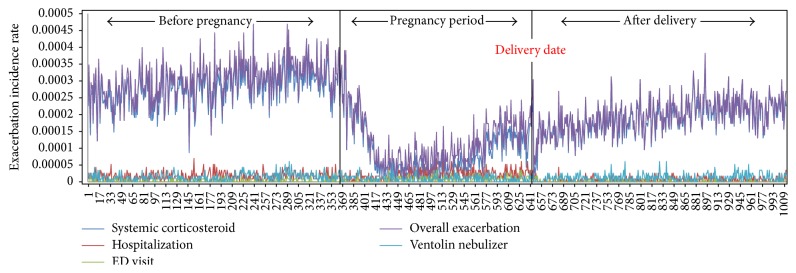
The overall asthma exacerbations were observed less during pregnancy than before and after it. Corticosteroid burst therapy showed a sharp fall at the beginning of pregnancy and slowly increased after delivery. None of the asthma-related hospitalizations, emergency department (ED) visits, and Ventolin nebulizer treatments at the outpatient clinic showed an increasing trend during pregnancy.

**Figure 6:**
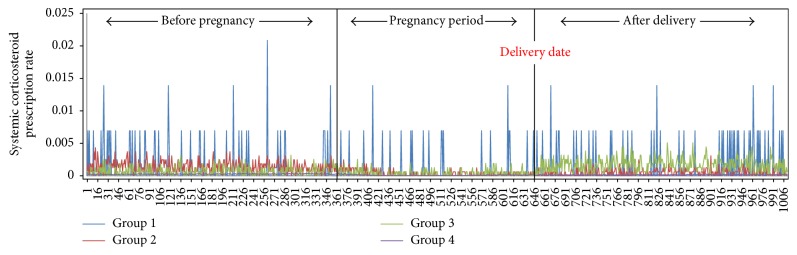
Use of systemic corticosteroids according to the study groups. Corticosteroid burst therapy showed a sharp fall at the beginning of pregnancy and slowly increased after delivery across the 4 groups.

Table 4 shows the overall frequencies of variables related to asthma exacerbations. Hospitalization and ED visits were significantly increased during pregnancy (*P* < 0.001); otherwise, overall exacerbations and systemic corticosteroids were decreased (*P* < 0.001) (Figure 5). Asthma-related hospitalizations and ED visits increased in Groups 1 and 3 during pregnancy (*P* < 0.001). However the other groups did not show any change in trend. Group 1 showed similar patterns of overall exacerbations (*P* = 0.476), systemic corticosteroids (*P* = 0.173), ED visits (*P* = 0.569), and Ventolin nebulizer treatments at the outpatient clinic (*P* = 0.466) during pregnancy, compared to before and after it. Corticosteroid bursts showed a sharp fall at the beginning of pregnancy and slowly increased after delivery (Figure 6). Group 3 shows gradual increases of asthma exacerbations before the pregnancy but there are no asthma exacerbations during the pregnancy. However after the pregnancy, the amount of asthma exacerbations sharply increases and it may be related to poor adherence to asthma medication during pregnancy.

### 3.4. Outpatient Clinic Utilization before, during, and after Pregnancy

Outpatient clinic utilization according to specialty was calculated as the number of visits to each specialty versus the total number of outpatient visits. Overall outpatient utilization was significantly lower during pregnancy than during the other time periods. As expected, utilization of the obstetrics specialty increased significantly during pregnancy. Meanwhile, utilization of other specialties, in particular internal medicine, showed a significant decrease during pregnancy (*P* < 0.001) (Table 5).

### 3.5. Associations between the Rank-Sum Values of Asthma Medications and Asthma Exacerbations

Tables 6–9 show the results based on a quasi-Poisson regression in each group. Except for Group 4, the coefficients of* X*_*i*1_ (total use of asthma medications before pregnancy) were significantly negative, which implies that asthma exacerbations during pregnancy tended to be reduced as a greater number of medications were prescribed before pregnancy in Groups 2 and 3. However, in Group 4, the coefficients of* X*_*i*1_ in systemic corticosteroid prescriptions, ED visits, and overall exacerbations were positive. Moreover, there was no significant association in Group 1 between asthma exacerbations and the total usage of medication over time. In all groups except Group 1, the coefficients of* X*_*i*2_ (total use of asthma medications during pregnancy) were significantly positive, indicating that subjects with a higher level of medication during pregnancy tended to have more asthma exacerbations. In Groups 3 and 4, there were positive associations between* X*_*i*3_ (total use of asthma medications after pregnancy) and asthma exacerbations. Finally, the proportions of subjects with asthma exacerbations during pregnancy could be explained by the level of asthma medications during the time periods before and during pregnancy (Table 10).

### 3.6. Annual Prevalence of Asthma during Pregnancy

The annual prevalence of asthma during pregnancy was 4.48% (95% CI, 4.42%–4.54%) in 2011, 4.61% (95% CI, 4.55%–4.67%) in 2012, and 4.97% (95% CI, 4.91%–5.04%) in 2013. No significant trend was observed through the years (*p* = 0.30).

## 4. Discussion

We have presented quantitative rank-sum values of asthma medications and their correlation with asthma exacerbations during pregnancy and compared the effect of pregnancy on the maintenance of asthma medications and asthma exacerbation during pregnancy to that before and after pregnancy. This study was conducted as a nationwide population-based retrospective cohort study using the HIRA database and included 115,169 asthmatic pregnant women. Our study showed that qualitative and quantitative asthma medications were reduced in most asthmatics during pregnancy.

It is well known that approximately one-third of asthma patients naturally improve during pregnancy. Similarly, in our study, we found that some of the asthmatic pregnant women did improve. Conversely, some of the patients experienced a worsened level of control during pregnancy. Among 115,169 asthmatic pregnant women, all subjects were clustered into four groups: severe persistent (*n* = 225, 0.19%), mild persistent (*n* = 108,725, 94.4%), worsened (*n* = 2,968, 2.58%), and improved (*n* = 3,251, 2.82%) asthma during pregnancy. The patterns of asthma medication use and their correlation with asthma exacerbation differed according to the study groups. Interestingly among our clustered groups, subjects in Group 3 tend to avoid asthma medication during pregnancy. However, there was no overall correlation between reduced asthma medications and asthma exacerbations during pregnancy in each group.

We hypothesized that a reduction of asthma medication during pregnancy might be correlated with asthma exacerbations. However, our findings showed the opposite, where a higher level of asthma medication use tended to produce more asthma exacerbations in each group. The results suggested that asthma exacerbations depend more on the baseline severity of asthma and its natural course during pregnancy than on adherence to asthma medication use if group effects are controlled. Therefore, it is clinically significant that individualized therapeutic strategies are utilized, including closed monitoring, and the active control of susceptible pregnant asthma patients before and during pregnancy is warranted.

Many studies have demonstrated that pregnant women preferred to use ICS monotherapy rather than oral or combination asthma medications due to concerns regarding systemic adverse effects [11, 12]. A recent study in seven European regions reported that the overall prescription of oral asthma medications, such as oral prednisolone and LTRAs, was reduced during pregnancy and an interpretation of their results suggested that LTRAs should not be started during pregnancy but could continue in women who were already using them for the successful control of their asthma before pregnancy [12]. Systemic corticosteroids and high-dose ICS are known to increase the risk of preeclampsia, low birth weight, and preterm delivery [33, 34]. Although several studies and guidelines have emphasized that an adequate dose of ICS and SABA did not affect pregnancy outcomes [6, 35], pregnant women have been reluctant to take steroids during pregnancy due to safety concerns. Our findings also support previous results that South Korean asthmatic pregnant women preferred ICS-based inhalers over oral asthma medications regardless of the severity of their asthma and the level of asthma medications before pregnancy [7]. In reality, the US and Korean Food and Drug Administration put most asthma medications, even inhalers, into category C. Achieving well-controlled asthma will greatly reduce the need for high-dose ICSs or systemic corticosteroids and also prevent the risk of adverse pregnancy or perinatal outcomes [33].

The strengths of the current study are as follows: this study was a nationwide cohort study with a large sample size. The HIRA data used in the current study included all information with respect to hospital stays as well as ED visits and outpatient utilization. Previous studies conducted in Europe have noted that a lack of information on hospital stays was an inherent limitation of their studies [11, 12]. Indeed, information on hospital stays would be extremely valuable in exploring the reduction of asthma medications and their association with asthma symptoms. Moreover, quantitative analysis using the rank-sum values of asthma medications and clustering of groups according to the rank-sum values of asthma medications would be a valuable approach. All of the previous studies have analyzed a rate or frequency of asthma medication at the group level [7, 11, 12, 14]. However, we considered the daily rank-sum values of asthma medications at the individual level and categorized all subjects into four groups, namely, severe persistent, mild persistent, worsened, and improved asthma during pregnancy. We found that the patterns of asthma medication use and their correlation with asthma exacerbations differed according to the study groups. Furthermore, subjects in Group 3 tend to avoid asthma medication during pregnancy.

Our study had several limitations. First, while we were able to find accurate records regarding the prescription and dispensing of asthma medications, this did not guarantee that patients actually used the drugs. In reality, this is an inherent limitation of healthcare database analysis. To overcome this limitation, a well-designed prospective cohort study should be considered. Second, the HIRA data did not contain objective measurements including lung function tests or bronchial provocation tests reflecting a confirmed asthma diagnosis and asthma severity. It also did not include subjective measurements such as the asthma control test to reflect asthma symptoms. For these reasons, the HIRA data did not guarantee an accurate diagnosis of asthma and determination of asthma exacerbations. The prevalence of asthma during pregnancy was approximately 8.7% in our study, which is consistent with the previous findings. Taking these findings together, we hypothesized that asthmatic pregnant women tended to discontinue asthma medication and that this factor was related to increased asthma exacerbations. Oral asthma medications and combination inhalers rather than inhaler corticosteroids were stopped from the beginning through to the end of pregnancy. Increased asthma exacerbations were found only in a small part of the cohort despite an overall reduction in the use of asthma medications. Owing to the limitation of the HIRA data, we failed to conclude whether reduced asthma exacerbations were derived from an improvement of asthma, avoidance of healthcare utilization, or ignoring symptoms due to concerns regarding medication-related adverse effects on their fetus.

In conclusion, asthmatic pregnant women showed a trend for stopping asthma medication early on in pregnancy and showed more asthma exacerbations in the subpopulations. These results have an important implication in the management of asthmatic pregnant women. Further research on the safety of asthma medication during pregnancy and guideline-based education emphasizing the importance of maintenance therapy during pregnancy will be essential.

## Figures and Tables

**Table 1:** Ranking of asthma medication according to the guideline-based stepwise approach.

Component	Type	Rank	Prescription period
LTRAs			
Montelukast	Oral	1	1
Pranlukast	Oral	1	1
Zafirlukast	Oral	1	1
Xanthine			
Aminophylline	Oral	1	1
Theophylline	Oral	1	1
LABA			
Bambuterol	Oral	1	1
Fenoterol	Oral	1	1
Formoterol	Oral	1	1
Procaterol	Oral	1	1
Procaterol	Inhaler	1	30
Terbutaline	Oral	1	1
ICS			
Low-dose ICS			
Budesonide	Inhaler	1	30
Ciclesonide	Inhaler	1	30
Fluticasone propionate	Inhaler	1	30
Medium- to high-dose ICS			
Budesonide	Inhaler	2	30
Fluticasone propionate	Inhaler	2	30
ICS/LABA			
Low-dose ICS/LABA			
Budesonide/formoterol	Inhaler	2	30
Budesonide/formoterol^*∗*^	Inhaler	2	60^*∗*^
Beclomethasone dipropionate/formoterol	Inhaler	2	30
Fluticasone furoate/vilanterol	Inhaler	2	30
Fluticasone propionate/formoterol	Inhaler	2	30
Fluticasone propionate/salmeterol	Inhaler	2	30
Fluticasone propionate/salmeterol^*∗∗*^	Inhaler	2	14^*∗∗*^
Medium- to high-dose ICS/LABA			
Budesonide/formoterol	Inhaler	3	30
Fluticasone propionate/salmeterol	Inhaler	3	30
Fluticasone propionate/salmeterol^*∗∗*^	Inhaler	3	14^*∗∗*^
Fluticasone propionate/formoterol	Inhaler	3	30
Fluticasone furoate/vilanterol	Inhaler	3	30
LAMA			
Tiotropium	Inhaler	4	30
Systemic corticosteroids			
Betamethasone < 2.4 mg		4	1
Dexamethasone < 3 mg		4	1
Methylprednisolone < 16 mg		4	1
Prednisolone < 20 mg		4	1

^*∗*^The device contains 120 doses, twice the dose of the standard device. ^*∗∗*^The device contains 28 doses for use over 14 days; LTRA, leukotriene receptor antagonist; LABA, long-acting *β*_2_-agonists; ICS, inhaled corticosteroid; LAMA, long-acting muscarinic antagonists.

**Table 2:** Comparison of oral asthma medications before, during, and after pregnancy.

Medication	Group	Before pregnancy	During pregnancy	After pregnancy	
		Days/month			
		Mean ± sd	Mean ± sd	Mean ± sd	*P* ^*∗*^
LTRAs	Total	0.229 ± 1.82	0.092 ± 1.24	0.189 ± 1.71	<0.0001
	Group 1	3.804 ± 9.33	3.054 ± 8.35	4.083 ± 9.52	0.0003
	Group 2	1.946 ± 6.45	0.852 ± 4.23	0.97 ± 4.22	<0.0001
	Group 3	0.925 ± 4.21	0.568 ± 3.58	1.477 ± 5.93	<0.0001
	Group 4	0.151 ± 1.21	0.05 ± 0.75	0.122 ± 1.15	<0.0001

Xanthine	Total	0.05 ± 0.68	0.017 ± 0.47	0.028 ± 0.57	<0.0001
	Group 1	1.684 ± 6.45	1.592 ± 6.26	1.43 ± 5.79	0.3094
	Group 2	0.26 ± 2.07	0.101 ± 1.32	0.114 ± 1.14	<0.0001
	Group 3	0.127 ± 1.2	0.64 ± 1.11	0.205 ± 2.05	<0.0001
	Group 4	0.038 ± 0.48	0.01 ± 0.25	0.018 ± 0.34	<0.0001

LABA	Total	0.112 ± 0.86	0.032 ± 0.47	0.07 ± 0.71	<0.0001
	Group 1	0.793 ± 3.86	0.249 ± 1.94	0.402 ± 2.46	<0.0001
	Group 2	0.318 ± 2.01	0.088 ± 1.11	0.229 ± 1.53	<0.0001
	Group 3	0.225 ± 1.42	0.059 ± 0.938	0.165 ± 1.58	<0.0001
	Group 4	0.102 ± 0.75	0.029 ± 0.41	0.062 ± 0.61	<0.0001

Low-dose systemic corticosteroids	Total	0.431 ± 1.83	0.116 ± 1.21	0.313 ± 1.73	<0.0001
	Group 1	14.128 ± 14.13	13.808 ± 13.4	15.657 ± 14.25	<0.0001
	Group 2	2.015 ± 4.77	0.462 ± 2.51	1.04 ± 3.01	<0.0001
	Group 3	1.29 ± 3.21	0.36 ± 2.53	1.169 ± 4.61	<0.0001
	Group 4	0.331 ± 1.29	0.071 ± 0.643	0.236 ± 1.17	<0.0001

^*∗*^ *P* value according to the multivariate analysis of variance; LTRA, leukotriene receptor antagonist; LABA, long-acting *β*_2_-agonists; sd, standard deviation.

**Table 3:** Comparison of inhaled asthma medications before, during, and after pregnancy, according to the study groups.

Medication	Group	Before pregnancy	During pregnancy	After pregnancy	
		Doses/month			
		Mean ± sd	Mean ± sd	Mean ± sd	*P* ^*∗*^
Low-dose ICS	Total	0.0013 ± 0.047	0.0031 ± 0.079	0.0008 ± 0.038	<0.0001
	Group 1	0.0204 ± 0.19	0.0631 ± 0.353	0.0111 ± 0.156	<0.0001
	Group 2	0.0175 ± 0.177	0.0434 ± 0.294	0.0082 ± 0.123	<0.0001
	Group 3	0.0105 ± 0.135	0.0277 ± 0.243	0.0112 ± 0.149	<0.0001
	Group 4	0.0005 ± 0.028	0.001 ± 0.046	0.0003 ± 0.02	<0.0001

Medium- to high-dose ICS	Total	0.0009 ± 0.041	0.0029 ± 0.077	0.0006 ± 0.035	<0.0001
	Group 1	0.0207 ± 0.191	0.0636 ± 0.355	0.011 ± 0.157	<0.0001
	Group 2	0.0146 ± 0.169	0.042 ± 0.291	0.0069 ± 0.118	<0.0001
	Group 3	0.0086 ± 0.127	0.0277 ± 0.243	0.0104 ± 0.146	<0.0001
	Group 4	0.0002 ± 0.201	0.001 ± 0.043	0.0002 ± 0.017	<0.0001

Low-dose ICS/LABA	Total	0.0057 ± 0.081	0.0045 ± 0.073	0.004 ± 0.069	<0.0001
	Group 1	0.1926 ± 0.527	0.176 ± 0.485	0.1715 ± 0.48	0.2656
	Group 2	0.09 ± 0.315	0.056 ± 0.25	0.0426 ± 0.21	<0.0001
	Group 3	0.041 ± 0.211	0.387 ± 0.213	0.0588 ± 0.264	<0.0001
	Group 4	0.0019 ± 0.044	0.0017 ± 0.042	0.001 ± 0.033	<0.0001

Medium- to high-dose ICS/LABA	Total	0.0007 ± 0.036	0.0006 ± 0.032	0.0005 ± 0.032	0.0005
	Group 1	0.0937 ± 0.421	0.0769 ± 0.375	0.0744 ± 0.367	0.1463
	Group 2	0.0127 ± 0.155	0.0069 ± 0.114	0.003 ± 0.071	<0.0001
	Group 3	0.0039 ± 0.084	0.0057 ± 0.104	0.0107 ± 0.143	<0.0001
	Group 4	0.00004 ± 0.009	0.00006 ± 0.01	0.00003 ± 0.008	0.0273

SABAs	Total	0.0043 ± 0.078	0.0034 ± 0.067	0.0032 ± 0.037	<0.0001
	Group 1	0.1059 ± 0.375	0.1053 ± 0.395	0.1081 ± 0.431	0.9656
	Group 2	0.0352 ± 0.21	0.0304 ± 0.196	0.0281 ± 0.192	<0.0001
	Group 3	0.0192 ± 0.154	0.0192 ± 0.164	0.0202 ± 0.171	0.6736
	Group 4	0.0027 ± 0.064	0.002 ± 0.05	0.0018 ± 0.05	<0.0001

LAMA	Total	0.00004 ± 0.002	0.00001 ± 0.002	0.00002 ± 0.005	<0.0001
	Group 1	0.00037 ± 0.019	0.0013 ± 0.036	0.0056 ± 0.079	0.0006
	Group 2	0.00005 ± 0.007	0	0.00026 ± 0.005	0.4227
	Group 3	0.00003 ± 0.005	0.0001 ± 0.01	0.00036 ± 0.019	0.0014
	Group 4	0.00001 ± 0.001	0	0.00002 ± 0.001	0.4227

^*∗*^ *P* value according to the multivariate analysis of variance; ICS, inhaled corticosteroid; LABA, long-acting *β*_2_-agonists; SABAs, short-acting *β*_2_-agonists; LAMA, long-acting muscarinic antagonists; sd, standard deviation.

**Table 4:** Comparison of asthma exacerbations before, during, and after pregnancy, according to the study groups.

Exacerbation	Group	Before pregnancy	During pregnancy	After pregnancy	
		Frequencies/month			
		Mean ± sd	Mean ± sd	Mean ± sd	*P* ^*∗*^
Systemic corticosteroid prescription	Total	0.0081 ± 0.116	0.0027 ± 0.059	0.0054 ± 0.096	<0.0001
	Group 1	0.0307 ± 0.195	0.0293 ± 0.211	0.04 ± 0.273	0.1703
	Group 2	0.0205 ± 0.213	0.0073 ± 0.097	0.0142 ± 0.156	<0.0001
	Group 3	0.0222 ± 0.22	0.0057 ± 0.088	0.0117 ± 0.169	<0.0001
	Group 4	0.0072 ± 0.107	0.0025 ± 0.056	0.0048 ± 0.09	<0.0001

Hospitalization	Total	0.0005 ± 0.023	0.0007 ± 0.028	0.0002 ± 0.015	<0.0001
	Group 1	0.0074 ± 0.094	0.0164 ± 0.137	0.007 ± 0.084	0.0023
	Group 2	0.003 ± 0.056	0.0052 ± 0.076	0.0012 ± 0.034	<0.0001
	Group 3	0.0012 ± 0.035	0.0045 ± 0.073	0.0015 ± 0.041	<0.0001
	Group 4	0.0004 ± 0.021	0.0004 ± 0.021	0.0001 ± 0.012	<0.0001

ED visit	Total	0.0001 ± 0.01	0.0002 ± 0.016	0.00004 ± 0.007	<0.0001
	Group 1	0.0033 ± 0.064	0.0049 ± 0.082	0.003 ± 0.054	0.5691
	Group 2	0.0009 ± 0.031	0.0022 ± 0.05	0.0003 ± 0.018	<0.0001
	Group 3	0.0003 ± 0.016	0.0018 ± 0.044	0.0006 ± 0.025	<0.0001
	Group 4	0.0001 ± 0.008	0.0001 ± 0.011	0.00002 ± 0.004	<0.0001

Overall exacerbation	Total	0.0087 ± 0.121	0.0037 ± 0.075	0.0056 ± 0.099	<0.0001
	Group 1	0.0415 ± 0.272	0.0507 ± 0.335	0.0504 ± 0.323	0.4758
	Group 2	0.0289 ± 0.235	0.0146 ± 0.167	0.0157 ± 0.165	<0.0001
	Group 3	0.0236 ± 0.228	0.0119 ± 0.161	0.0138 ± 0.184	<0.0001
	Group 4	0.0077 ± 0.111	0.003 ± 0.065	0.005 ± 0.092	<0.0001

Ventolin nebulizer treatment	Total	0.0032 ± 0.061	0.003 ± 0.059	0.0026 ± 0.057	<0.0001
	Group 1	0.0967 ± 0.347	0.0844 ± 0.328	0.0904 ± 0.363	0.4664
	Group 2	0.0333 ± 0.199	0.0293 ± 0.189	0.0259 ± 0.177	<0.0001
	Group 3	0.0175 ± 0.144	0.0181 ± 0.157	0.0187 ± 0.159	0.5906
	Group 4	0.0018 ± 0.044	0.0016 ± 0.041	0.0012 ± 0.038	<0.0001

^*∗*^ *P* value according to the multivariate analysis of variance; ED, emergency department; sd, standard deviation.

**Table 5:** Comparison of outpatient clinic visits before, during, and after pregnancy, according to the study groups.

Exacerbation	Group	Before pregnancy	During pregnancy	After pregnancy	
		Visits/month			
		Mean ± sd	Mean ± sd	Mean ± sd	*P* ^*∗*^
Total number of outpatient visits	Total	1.1267 ± 2.467	1.9067 ± 2.411	1.1998 ± 2.876	<0.0001
	Group 1	2.2038 ± 6.667	2.9278 ± 6.313	2.2594 ± 6.344	0.8767
	Group 2	1.9313 ± 4.51	2.3157 ± 4.86	1.8011 ± 4.387	0.2158
	Group 3	1.6899 ± 3.89	2.091 ± 4.54	1.3879 ± 4.19	0.3454
	Group 4	1.1287 ± 2.471	1.8867 ± 2.388	1.1576 ± 2.612	0.1345

Internal medicine	Total	0.4312 ± 1.934	0.2178 ± 1.813	0.4062 ± 1.864	<0.0001
	Group 1	1.0946 ± 5.412	1.0156 ± 5.167	1.1599 ± 5.678	0.3154
	Group 2	0.8176 ± 2.972	0.4467 ± 2.34	0.6678 ± 2.871	<0.0001
	Group 3	0.6479 ± 2.72	0.3196 ± 2.14	0.5137 ± 2.64	<0.0001
	Group 4	0.4579 ± 1.595	0.2189 ± 1.648	0.3846 ± 1.754	<0.0001

Obstetrics and gynecology	Total	0.2167 ± 1.534	1.4376 ± 3.034	0.2676 ± 1.76	<0.0001
	Group 1	0.3066 ± 2.879	1.4567 ± 5.166	0.2675 ± 2.433	<0.0001
	Group 2	0.2345 ± 1.872	1.4699 ± 3.84	0.2788 ± 2.103	<0.0001
	Group 3	0.2387 ± 1.878	1.4678 ± 3.416	0.2647 ± 2.037	<0.0001
	Group 4	0.2134 ± 1.16	1.4122 ± 2.768	0.2478 ± 1.652	<0.0001

General practitioner	Total	0.0016 ± 0.049	0.0012 ± 0.027	0.0019 ± 0.051	<0.0001
	Group 1	0.0213 ± 0.541	0.0027 ± 0.161	0.0008 ± 0.071	0.2134
	Group 2	0.0027 ± 0.073	0.0014 ± 0.054	0.0026 ± 0.067	0.0154
	Group 3	0.0024 ± 0.068	0.0019 ± 0.061	0.0022 ± 0.063	<0.0001
	Group 4	0.0017 ± 0.042	0.0015 ± 0.038	0.0019 ± 0.04	<0.0001

General surgery	Total	0.0307 ± 0.713	0.0208 ± 0.498	0.0312 ± 0.671	<0.0001
	Group 1	0.05 ± 0.78	0.017 ± 0.064	0.0421 ± 0.59	0.2783
	Group 2	0.0374 ± 0.201	0.0233 ± 0.068	0.0419 ± 0.204	<0.0001
	Group 3	0.0337 ± 0.179	0.0217 ± 0.22	0.0347 ± 0.483	<0.0001
	Group 4	0.0307 ± 0.462	0.0201 ± 0.216	0.0318 ± 0.475	<0.0001

Otolaryngology	Total	0.1678 ± 1.57	0.0617 ± 0.514	0.1248 ± 0.876	<0.0001
	Group 1	0.2097 ± 2.376	0.137 ± 1.164	0.1724 ± 1.311	0.1674
	Group 2	0.2648 ± 2.186	0.1301 ± 1.152	0.1924 ± 0.954	<0.0001
	Group 3	0.2467 ± 2.067	0.0846 ± 0.867	0.1154 ± 0.941	<0.0001
	Group 4	0.1597 ± 2.276	0.0678 ± 0.531	0.1038 ± 0.775	<0.0001

Family medicine	Total	0.0237 ± 0.872	0.0084 ± 0.103	0.0211 ± 0.612	<0.0001
	Group 1	0	0.017 ± 0.51	0.0005 ± 0.003	<0.0001
	Group 2	0.0357 ± 0.193	0.013 ± 0.167	0.0007 ± 0.008	<0.0001
	Group 3	0.0287 ± 0.178	0.0107 ± 0.158	0.0004 ± 0.005	<0.0001
	Group 4	0.0248 ± 0.387	0.0072 ± 0.113	0.0218 ± 0.412	<0.0001

^*∗*^ *P* value according to the multivariate analysis of variance.

**Table 6:** General linear mixed models to estimate asthma medication pattern variables over the study period to predict asthma exacerbation in Group 1.

Outcome variable	Explanatory variable	Estimate	Standard Error	DF	*t*-value	*P* value	95% CI	
							Lower	Upper
Systemic corticosteroid prescription	Intercept	−0.9301	0.2563	222	−3.63	0.0004	−1.4324	−0.4278
	*X*_*i*1_	−0.00036	0.000563	222	−0.64	0.5242	−0.0015	0.00074
	*X*_*i*2_	−0.00036	0.000698	222	−0.51	0.6072	−0.0017	0.00101

Hospitalization	Intercept	−1.9751	0.2567	222	−7.69	<0.0001	−2.4782	−1.472
	*X*_*i*1_	−0.00075	0.000542	222	−1.38	0.1683	−0.0018	0.00031
	*X*_*i*2_	0.0001	0.00071	222	0.14	0.8878	−0.0013	0.00149

ED visit	Intercept	−3.2084	0.4634	222	−6.92	<0.0001	−4.1167	−2.3001
	*X*_*i*1_	−0.00078	0.000929	222	−0.84	0.403	−0.0026	0.00104
	*X*_*i*2_	0.000964	0.001373	222	0.7	0.4832	−0.0017	0.00366

Overall exacerbation	Intercept	−0.5459	0.2238	222	−2.44	0.0155	−0.9845	−0.1073
	*X*_*i*1_	−0.00049	0.000488	222	−1	0.3197	−0.0014	0.00047
	*X*_*i*2_	−0.00016	0.000618	222	−0.26	0.7956	−0.0014	0.00105

Ventolin nebulizer treatment	Intercept	−1.9872	0.5328	222	−3.73	0.0002	−3.0315	−0.9429
	*X*_*i*1_	−0.00073	0.001117	222	−0.66	0.5126	−0.0029	0.00146
	*X*_*i*2_	−0.00032	0.0014	222	−0.23	0.8167	−0.0031	0.00242

*X*_*i*1_, total use of asthma medications before pregnancy; *X*_*i*2_, total use of asthma medications during pregnancy; ED, emergency department; DF, degree of freedom; CI, confidence interval.

**Table 7:** General linear mixed models to estimate asthma medication pattern variables over the study period to predict asthma exacerbation in Group 2.

Outcome variable	Explanatory variable	Estimate	Standard Error	DF	*t*-value	*P* value	95% CI	
							Lower	Upper
Systemic corticosteroid prescription	Intercept	−2.801	0.09869	3248	−28.38	<0.0001	−2.9944	−2.6076
	*X*_*i*1_	−0.00172	0.000575	3248	−2.98	0.0029	−0.0028	−0.0006
	*X*_*i*2_	0.002588	0.000576	3248	4.5	<0.0001	0.00146	0.00372

Hospitalization	Intercept	−3.5395	0.1251	3248	−28.28	<0.0001	−3.7847	−3.2943
	*X*_*i*1_	−0.00332	0.00068	3248	−4.88	<0.0001	−0.0047	−0.002
	*X*_*i*2_	0.004318	0.000505	3248	8.55	<0.0001	0.00333	0.00531

ED visit	Intercept	−4.193	0.1568	3246	−26.75	<0.0001	−4.5003	−3.8857
	*X*_*i*1_	−0.00156	0.000976	3246	−1.6	0.1093	−0.0035	0.00035
	*X*_*i*2_	0.004638	0.000645	3246	7.2	<0.0001	0.00337	0.0059
	*X*_*i*3_	0.002408	0.001377	3246	1.75	0.0805	−0.0003	0.00511
	*X*_*i*1_ *∗X*_*i*2_	−0.000006	0.000003	3246	−1.87	0.061	−0.00001	0.0000003

Overall exacerbation	Intercept	−2.2493	0.08595	3248	−26.17	<0.0001	−2.4178	−2.0808
	*X*_*i*1_	−0.00232	0.000482	3248	−4.82	<0.0001	−0.0033	−0.0014
	*X*_*i*2_	0.003557	0.000412	3248	8.63	<0.0001	0.00275	0.00436

Ventolin nebulizer treatment	Intercept	−5.5041	0.4281	3248	−12.86	<0.0001	−6.3432	−4.665
	*X*_*i*1_	−0.00157	0.002348	3248	−0.67	0.5043	−0.0062	0.00303
	*X*_*i*2_	0.003411	0.002116	3248	1.61	0.1071	−0.0007	0.00756

*X*_*i*1_, total use of asthma medications before pregnancy; *X*_*i*2_, total use of asthma medications during pregnancy; *X*_*i*3_, total use of asthma medications after delivery; ED, emergency department; DF, degree of freedom; CI, confidence interval.

**Table 8:** General linear mixed models to estimate medication pattern variables over the study period to predict asthma exacerbation in Group 3.

Outcome variable	Explanatory variable	Estimate	Standard Error	DF	*t*-value	*P* value	95% CI	
							Lower	Upper
Systemic corticosteroid prescription	Intercept	−3.1987	0.1148	2963	−27.86	<0.0001	−3.4237	−2.9737
	*X*_*i*1_	−0.00086	0.000719	2963	−1.2	0.2298	−0.0023	0.00055
	*X*_*i*2_	0.003845	0.000404	2963	9.53	<0.0001	0.00305	0.00464
	*X*_*i*3_	0.001945	0.000288	2963	6.76	<0.0001	0.00138	0.00251
	*X*_*i*2_ *∗X*_*i*3_	−0.000004	0.000001	2963	−3.03	0.0024	−0.000006	−0.000001

Hospitalization	Intercept	−3.5983	0.1196	2965	−30.1	<0.0001	−3.8327	−3.3639
	*X*_*i*1_	−0.00112	0.00084	2965	−1.33	0.1825	−0.0028	0.00053
	*X*_*i*2_	0.004252	0.000341	2965	12.48	<0.0001	0.00358	0.00492

ED visit	Intercept	−4.8786	0.1756	2962	−27.78	<0.0001	−5.2228	−4.5344
	*X*_*i*1_	−0.00666	0.001862	2962	−3.58	0.0004	−0.0103	−0.003
	*X*_*i*2_	0.004306	0.000485	2962	8.88	<0.0001	0.00336	0.00526
	*X*_*i*3_	0.003262	0.0003	2962	10.89	<0.0001	0.00267	0.00385
	*X*_*i*2_ *∗X*_*i*3_	−0.000005	0.000001	2962	−3.8	0.0001	−0.000007	−0.000002
	*X*_*i*1_ *∗X*_*i*3_	0.000009	0.000003	2962	3.27	0.0011	0.000004	0.000015

Overall exacerbation	Intercept	−2.6632	0.09936	2963	−26.8	<0.0001	−2.8579	−2.4685
	*X*_*i*1_	−0.00364	0.001236	2963	−2.95	0.0033	−0.0061	−0.0012
	*X*_*i*2_	0.003166	0.000308	2963	10.28	<0.0001	0.00256	0.00377
	*X*_*i*3_	0.001941	0.000214	2963	9.06	<0.0001	0.00152	0.00236
	*X*_*i*1_ *∗X*_*i*3_	0.000004	0.000002	2963	1.94	0.0524	−0.00000004	0.000009

Ventolin nebulizer treatment	Intercept	−5.8844	0.383	2965	−15.36	<0.0001	−6.6351	−5.1337
	*X*_*i*1_	−0.00104	0.003013	2965	−0.35	0.7287	−0.0069	0.00487
	*X*_*i*2_	0.003631	0.001277	2965	2.84	0.0045	0.00113	0.00613

*X*_*i*1_, total use of asthma medications before pregnancy; *X*_*i*2_, total use of asthma medications during pregnancy; *X*_*i*3_, total use of asthma medications after delivery; ED, emergency department; DF, degree of freedom; CI, confidence interval.

**Table 9:** General linear mixed models to estimate medication pattern variables over the study period to predict asthma exacerbation in Group 4.

Outcome variable	Explanatory variable	Estimate	Standard Error	DF	*t*-value	*P* value	95% CI	
							Lower	Upper
Systemic corticosteroid prescription	Intercept	−3.8531	0.02787	109000	−138.25	<0.0001	−3.9077	−3.7985
	*X*_*i*1_	0.002234	0.000959	109000	2.33	0.0198	0.00035	0.00411
	*X*_*i*2_	0.01219	0.000543	109000	22.46	<0.0001	0.01113	0.01325
	*X*_*i*3_	0.00531	0.000797	109000	6.66	<0.0001	0.00375	0.00687
	*X*_*i*2_ *∗X*_*i*3_	−0.00004	0.000011	109000	−3.24	0.0012	−0.00006	−0.00002
	*X*_*i*1_ *∗X*_*i*3_	−0.00004	0.000022	109000	−1.94	0.0518	−0.00008	0.000003

Hospitalization	Intercept	−5.8122	0.05791	109000	−100.36	<0.0001	−5.9257	−5.6987
	*X*_*i*1_	−0.00116	0.002169	109000	−0.53	0.5938	−0.0054	0.00309
	*X*_*i*2_	0.01468	0.000772	109000	19.02	<0.0001	0.01317	0.01619
	*X*_*i*3_	0.01139	0.001043	109000	10.92	<0.0001	0.00935	0.01343
	*X*_*i*1_ *∗X*_*i*2_	0.000105	0.000023	109000	4.52	<0.0001	0.00006	0.00015
	*X*_*i*2_ *∗X*_*i*3_	−0.00003	0.000012	109000	−2.27	0.0235	−0.00005	−0.000006
	*X*_*i*1_ *∗X*_*i*3_	−0.00006	0.000032	109000	−1.91	0.0559	−0.0001	0.000003

ED visit	Intercept	−7.1008	0.1013	109000	−70.07	<0.0001	−7.2993	−6.9023
	*X*_*i*1_	0.000814	0.003466	109000	0.23	0.8143	−0.006	0.00761
	*X*_*i*2_	0.01465	0.001053	109000	13.91	<0.0001	0.01259	0.01671
	*X*_*i*3_	0.01126	0.001493	109000	7.55	<0.0001	0.00833	0.01419
	*X*_*i*1_ *∗X*_*i*2_	0.000139	0.000034	109000	4.14	<0.0001	0.00007	0.00021
	*X*_*i*1_ *∗X*_*i*3_	−0.00009	0.00005	109000	−1.7	0.0886	−0.0002	0.000008

Overall exacerbation	Intercept	−3.6883	0.02589	109000	−142.48	<0.0001	−3.739	−3.6376
	*X*_*i*1_	0.001349	0.000952	109000	1.42	0.1567	−0.0005	0.00321
	*X*_*i*2_	0.0128	0.000466	109000	27.5	<0.0001	0.01189	0.01371
	*X*_*i*3_	0.006794	0.000665	109000	10.21	<0.0001	0.00549	0.0081
	*X*_*i*1_ *∗X*_*i*2_	0.000046	0.000015	109000	3.09	0.002	0.000017	0.000075
	*X*_*i*2_ *∗X*_*i*3_	−0.00003	0.000008	109000	−3.36	0.0008	−0.00005	−0.00001
	*X*_*i*1_ *∗X*_*i*3_	−0.00005	0.000019	109000	−2.74	0.0062	−0.00009	−0.00001

Ventolin nebulizer treatment	Intercept	−6.5181	0.1807	109000	−36.07	<0.0001	−6.8723	−6.1639
	*X*_*i*1_	−0.01108	0.008464	109000	−1.31	0.1903	−0.0277	0.00551
	*X*_*i*2_	0.009296	0.004475	109000	2.08	0.0378	0.00053	0.01807

*X*_*i*1_, total use of asthma medications before pregnancy; *X*_*i*2_, total use of asthma medications during pregnancy; *X*_*i*3_, total use of asthma medications after delivery; ED, emergency department; DF, degree of freedom; CI, confidence interval.

**Table 10:** Possibility of asthma exacerbations during pregnancy explained by asthma medication use in the time period before and during pregnancy.

*X*_*i*1_	*X*_*i*2_	Possibility of exacerbations
Low	High	Likely
Low	Low	Possible
High	High	Possible
High	Low	Uncertain

*X*_*i*1_, total use of asthma medications before pregnancy; *X*_*i*2_, total use of asthma medications during pregnancy.

## Abbreviations

ED: Emergency department
GINA: Global Initiative for Asthma
HIRA: Health Insurance Review and Assessment Service
ICD: International Statistical Classification of Disease
ICS: Inhaled corticosteroids
ICS/LABA: Inhaled corticosteroids combined with inhaled long-acting *β*_2_-agonists
LABA: Long-acting *β*_2_-agonist
LAMA: Long-acting muscarinic antagonist
LTRA: Oral leukotriene receptor antagonists
SABA: Short-acting *β*_2_-agonist.
